# Bibliometric and visual analysis of machine learning-based research in acute kidney injury worldwide

**DOI:** 10.3389/fpubh.2023.1136939

**Published:** 2023-03-17

**Authors:** Xiang Yu, RiLiGe Wu, YuWei Ji, Zhe Feng

**Affiliations:** ^1^State Key Laboratory of Kidney Diseases, Department of Nephrology, Chinese People's Liberation Army General Hospital, Chinese People's Liberation Army Institute of Nephrology, National Clinical Research Center of Kidney Diseases, Beijing, China; ^2^Medical Big Data Research Center, Chinese People's Liberation Army General Hospital, Beijing, China

**Keywords:** machine learning, acute kidney injury, bibliometric analysis, model, critical care, hotspot

## Abstract

**Background:**

Acute kidney injury (AKI) is a serious clinical complication associated with adverse short-term and long-term outcomes. In recent years, with the rapid popularization of electronic health records and artificial intelligence machine learning technology, the detection rate and treatment of AKI have been greatly improved. At present, there are many studies in this field, and a large number of articles have been published, but we do not know much about the quality of research production in this field, as well as the focus and trend of current research.

**Methods:**

Based on the Web of Science Core Collection, studies reporting machine learning-based AKI research that were published from 2013 to 2022 were retrieved and collected after manual review. VOSviewer and other software were used for bibliometric visualization analysis, including publication trends, geographical distribution characteristics, journal distribution characteristics, author contributions, citations, funding source characteristics, and keyword clustering.

**Results:**

A total of 336 documents were analyzed. Since 2018, publications and citations have increased dramatically, with the United States (143) and China (101) as the main contributors. Regarding authors, Bihorac, A and Ozrazgat-Baslanti, T from the University of Florida have published 10 articles. Regarding institutions, the University of California (18) had the most publications. Approximately 1/3 of the publications were published in Q1 and Q2 journals, of which Scientific Reports (19) was the most prolific journal. Tomašev et al.'s study that was published in 2019 has been widely cited by researchers. The results of cluster analysis of co-occurrence keywords suggest that the construction of AKI prediction model related to critical patients and sepsis patients is the research frontier, and XGBoost algorithm is also popular.

**Conclusion:**

This study first provides an updated perspective on machine learning-based AKI research, which may be beneficial for subsequent researchers to choose suitable journals and collaborators and may provide a more convenient and in-depth understanding of the research basis, hotspots and frontiers.

## 1. Introduction

Acute kidney injury (AKI) is a clinical emergency that can be caused by a variety of etiologies and is associated with multiple of acute and chronic comorbidities; the global incidence rate of AKI is between 5 and 50% (1). Even mild AKI may lead to chronic kidney disease, and severe or recurrent events may lead to end-stage renal disease (2). Although the detection ate and treatment of AKI have improved considerably and research on its pathogenesis and pathophysiological processes has gradually intensified, the morbidity and mortality rates of AKI are still increasing year by year, thus causing considerable psychological, physiological, and economic burdens to hospitalized patients (3).

Machine learning is a major branch of artificial intelligence technology, and it is defined as the study of algorithms that use computer systems to learn from sample data and past experience, to effectively identify hidden variable associations in massive datasets, to classify objects by specific criteria, and to make predictions based on baseline features (4). Machine learning is rapidly becoming an integral part of data analysis tools in a wide range of medical applications. With the development of hardware and software, advanced machine learning frameworks such as deep neural networks are increasingly being used to process a series of biomedical datasets. In the context of kidney disease, especially AKI, machine learning is also setting off a technological revolution, and its main functions include early diagnosis and prediction, prognosis assessment, imaging assistance, and identification of new genomic sites (5–9). At present, research in this field has been increasing, with a large number of publications each year, and continues to explore new applications of machine learning methods for the innovation of AKI diagnosis and treatment models. However, the quality of scientific publications in this field, as well as the focus and trends of research, are not well-understood.

Bibliometric analysis refers to interdisciplinary science that uses mathematical and statistical methods to quantitatively analyze all knowledge carriers, this carrier mainly refers to books or medical journal articles (10). In particular, the application of information visualization technology can intuitively display the research development history, research status, research hotspots, and development trend of the theme (11, 12). At present, a number of bibliometric research attempts have been carried out in various disciplines, which not only realize the quality evaluation of individual studies or researchers by academic institutions, funding institutions and independent researchers but also illustrate new priorities and breakthroughs for further studies. Therefore, in this review, we aim to intuitively analyze the research status of machine learning-related AKI through bibliometric methods to put forward suggestions and further views.

## 2. Methods

### 2.1. Search strategy

We searched the Web of Science Citation Database from January 1, 2013, to October 15, 2022. The retrieval strategy was stated as (database= Web of Science core collection), (topic1 =“acute kidney injury” or “AKI” or “acute kidney failure” or “acute renal failure”), and (topic2 = “machine learning” or “Naive Bayes” or “Decision trees” or “Random forest” or “Support vector machines” or “Gradient boosting decision tree” or “Adaptive boosting” or “Extreme gradient boosting” or “Light gradient boosting machine” or “Categorical boosting” or “Generalized additive model” or “Artificial neural networks” or “Deep learning”), (type = article), (year published = 2013–2022), and (language = English). All electronic searches were conducted at the same time (15:14 BST on October 15, 2022) to avoid changes in citation rate as much as possible. After all identified articles were retrieved, the results were sorted using the option “Times cited”, which yielded a list of all the articles published in a specific journal ranked by citation number. All documents are exported in tab-delimited file format, and the records include the full bibliographic record and the cited references, saved in “.txt” format.

### 2.2. Study selection

**Figure 1:**
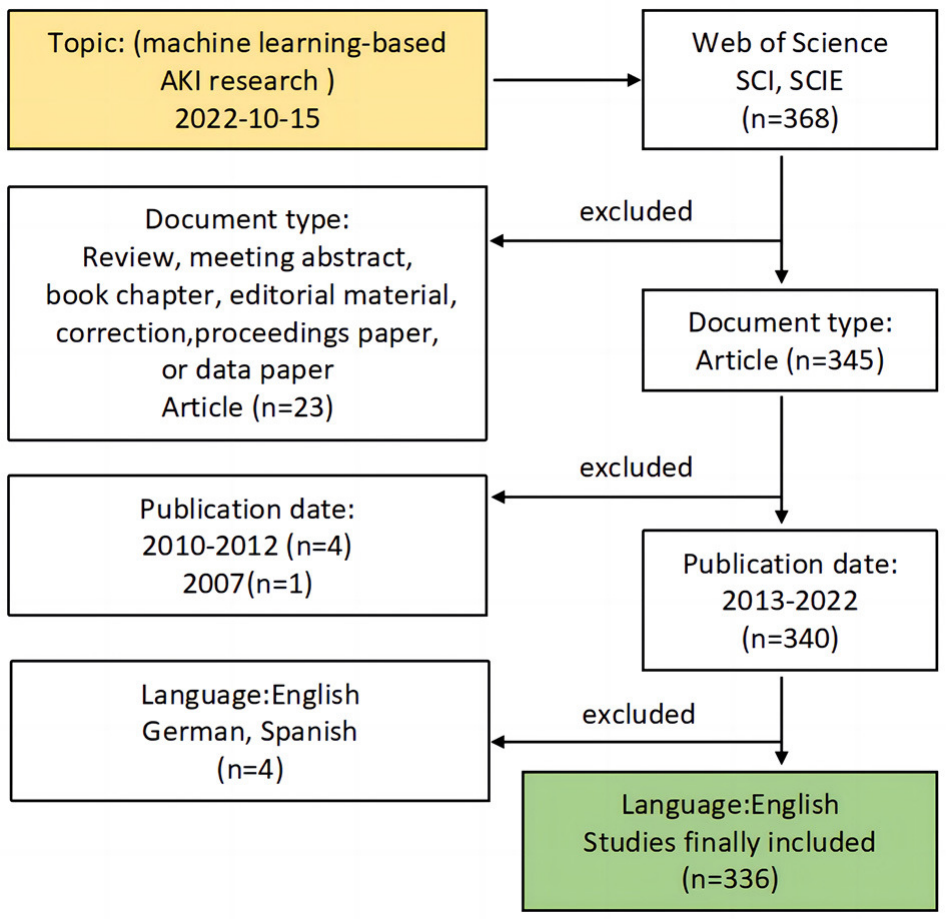
Flowchart of analyzed and excluded articles.

We selected all the retrieved literature and reviewed the abstracts by two independent nephrologists. Some of the literature was reviewed in detail by retrieving the complete original text from multiple databases, and a nephrologist was responsible for resolving any disagreements. We only included English literature, excluding literature in other languages, conference abstracts, science popularization and news reports (Figure 1).

### 2.3. Data processing and visual analysis

Co-cited articles, keywords, countries, institutions, journals, authors, and references were analyzed using VOSviewer software (Version.1.6.16, Center for Science and Technology Studies, University of Leiden, NLv.1.6.16). The H-index, impact factor (IF) and category quartile were collected from the Web of Science Citation Database. Microsoft Office Excel software (Version.2013, Microsoft Corporation, Redmond, WAS, USA) was used to analyze the data of publications, citations and polynomial trend lines, as well as the linear fitting analysis between the year and publication.

## 3. Results

### 3.1. Literature development trends

**Figure 2:**
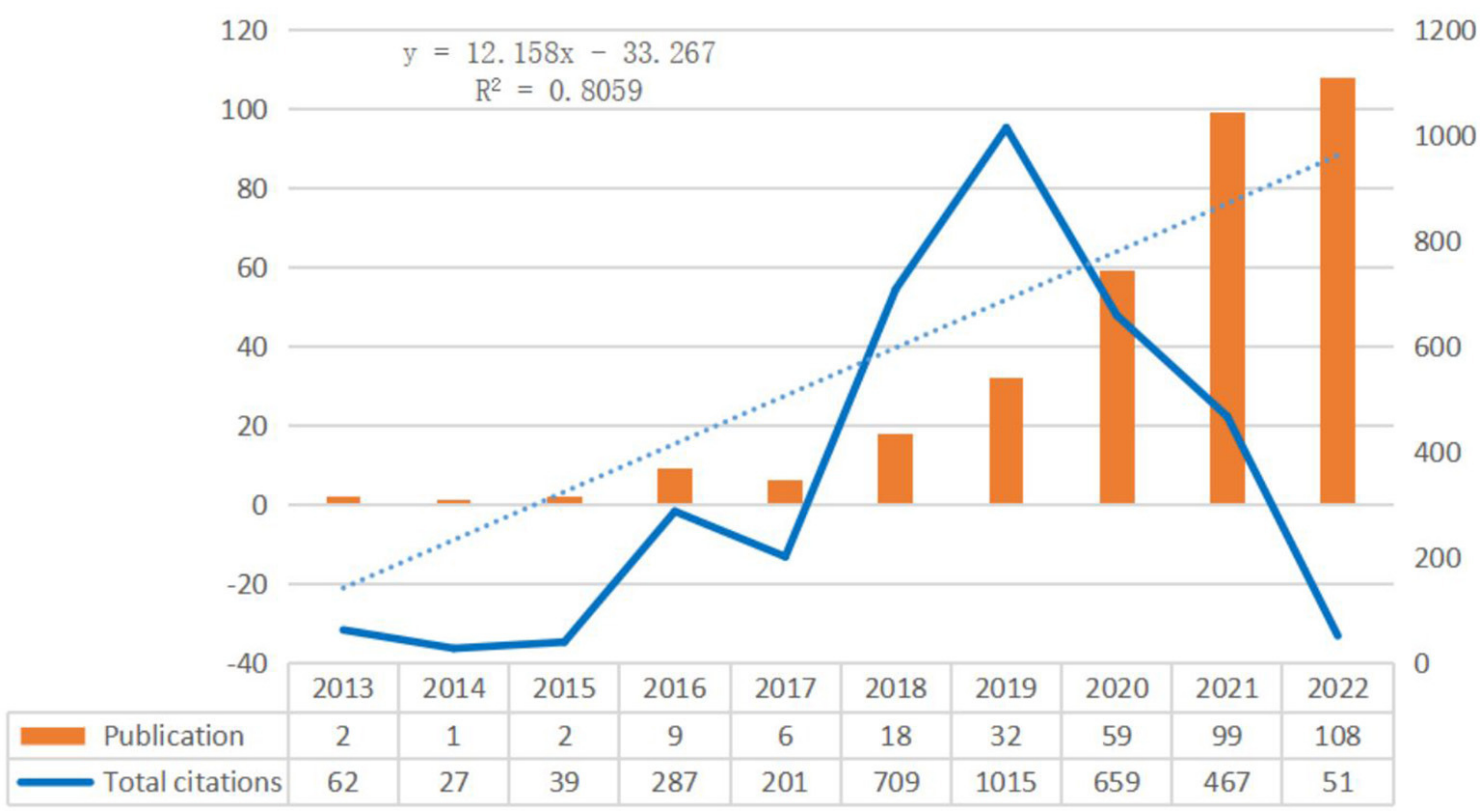
Trends in publications and citations of machine learning related AKI research.

Based on the Web of Science citation database, we retrieved a total of 336 English-language publications in the field of machine learning-based AKI research, with a total of 2,802 citations (excluding self-citations), a mean of 10.03 citations, and a total H-index of 28. The number of published articles rose sharply from 2018, and the number of published articles in 2022 was twice as high as that in 2020, representing nearly 30% of the total number of retrieved studies. In addition, since 2018, the number of citations has also increased year by year, and the total number of citations in 2019 was nearly 5 times that in 2016. The linear fitting analysis of all the included articles revealed a significant correlation between the year and publication (*R*^2^ = 0.8059), which represents increasing attention to this research field worldwide (Figure 2).

### 3.2. Geographical distribution characteristics

**Figure 3:**
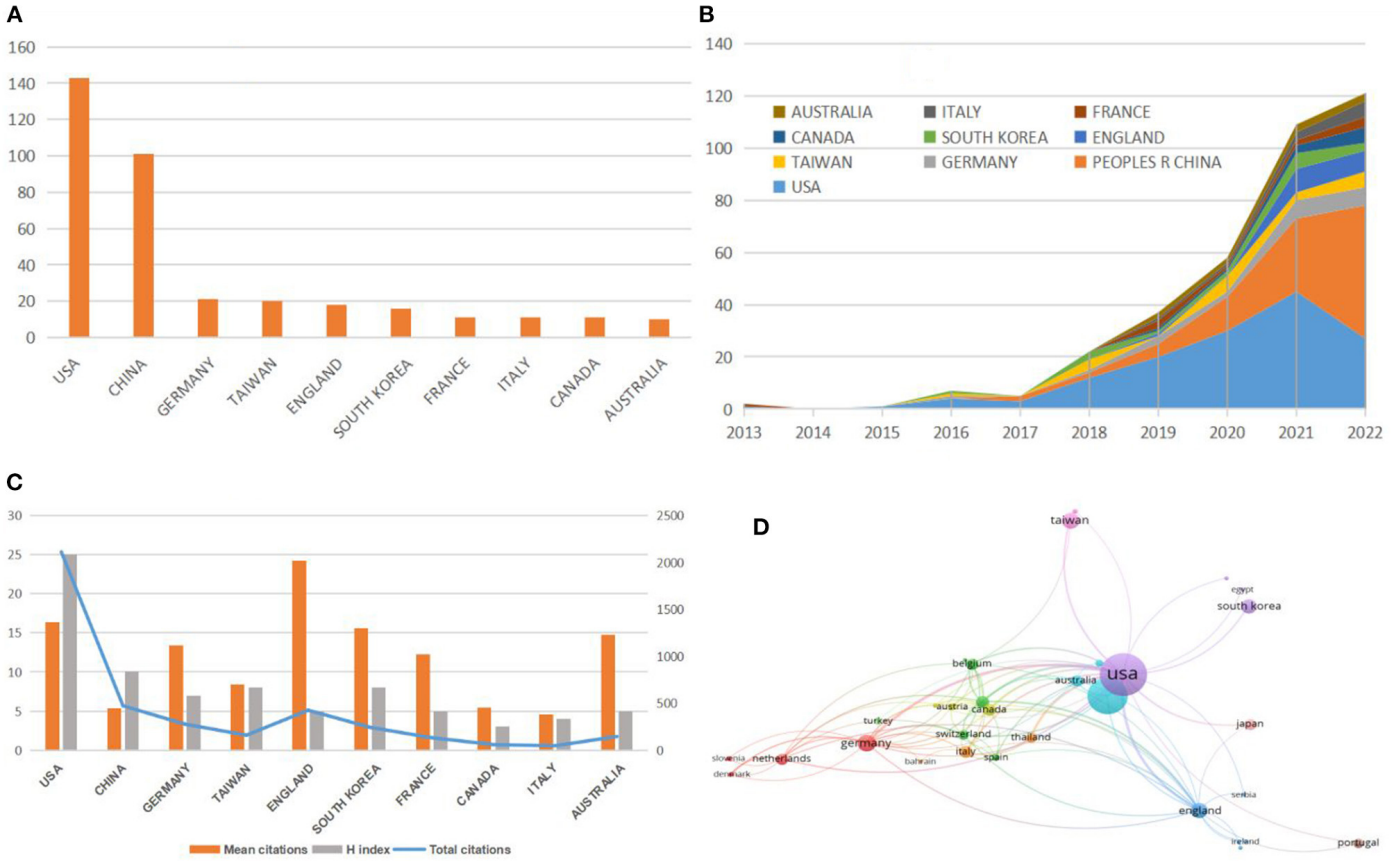
**(A)** Top 10 countries/regions by publications. **(B)** Temporal trends of publications from the top 10 countries/regions. **(C)** H-index, mean citations, and total citations of the top 10 countries/regions. **(D)** Collaboration network of countries/regions.

All literature was distributed among 45 countries/regions, including 702 institutions. The United States (143, 42.6%) produced the most publications, followed by China (101, 30.1%), Germany (21, 6.3%), Taiwan (20, 6.0%), and England (18, 5.4%; Table 1). The top twenty countries/regions are mainly concentrated in Asia and Europe, but the U.S. has more publications than the sum of the second, third, and fourth place countries (Figure 3A). The top five countries in terms of mean citations were England (24.22), Switzerland (22.75), the United Arab Emirates (20.5), the United States (16.4), and Austria (15.75). The top five countries in terms of H-index were the United States (25), China (10), South Korea (8), Taiwan (8), and Germany (7). These results demonstrated that machine learning-based AKI research had received widespread attention from global scholars, and the United States, China and some European countries were the leading contributors. In addition, the annual publications and citations of various countries are also analyzed (Figures 3B, C), and the United States represented a relatively closer cooperation in this research field (Figure 3D).

**Table 1:** Top 10 countries by publications, H-index, and citations.

**Rank**	**Country/region**	**Publications**	**Total citations**	**Mean citations**	**H-index**
1st	United States	143	2,111	16.4	25
2nd	China	101	473	5.3	10
3rd	Germany	21	278	13.43	7
4th	Taiwan	20	160	8.45	8
5th	England	18	426	24.22	5
6th	South Korea	16	244	15.63	8
7th	France	11	133	12.27	5
8th	Italy	11	47	4.55	4
9th	Canada	11	60	5.45	3
10th	Australia	10	146	14.7	5

**Figure 4:**
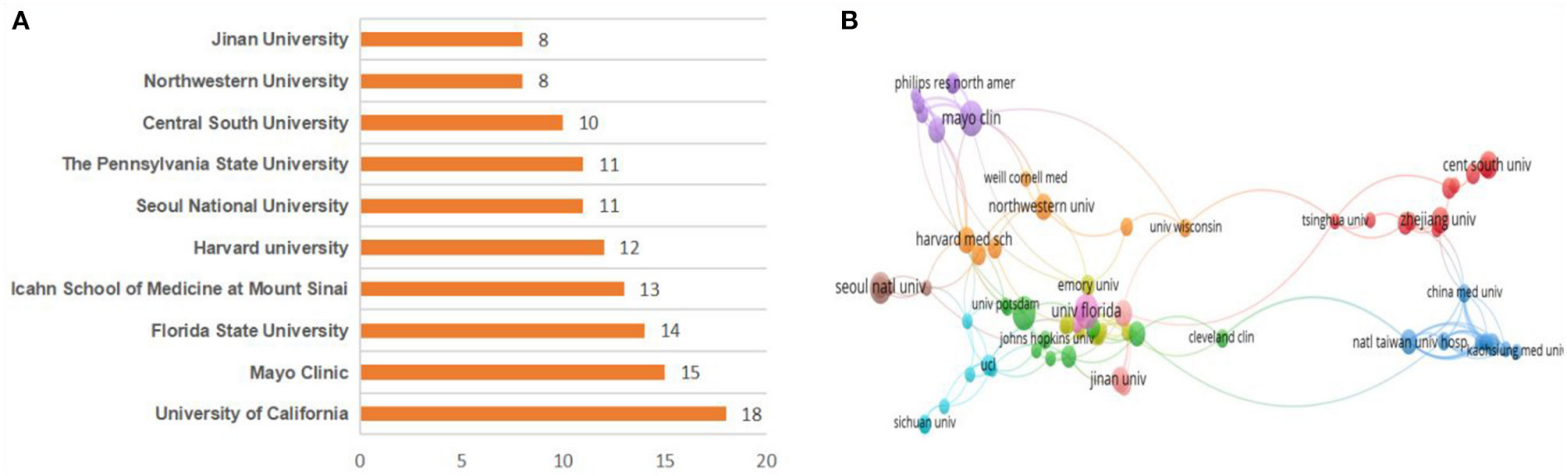
**(A)** Top 10 institutions by publications. **(B)** Collaboration network of institutions.

The top five institutions of publications were the University of California (18, 5.4%), the Mayo Clinic (15, 4.5%), Florida State University (14, 4.2%), the Icahn School of Medicine at Mount Sinai (13, 3.9%), and Harvard University (12, 3.6%). Seven of the top ten research institutions are located in the United States (Figure 4A). However, the interagency cooperation network diagram shows that the centrality value of each institution is low, indicating that the cooperation between them is not close enough, which may be related to the lower number of publications in this field (Figure 4B).

### 3.3. Contribution of authors and co-cited authors

**Figure 5:**
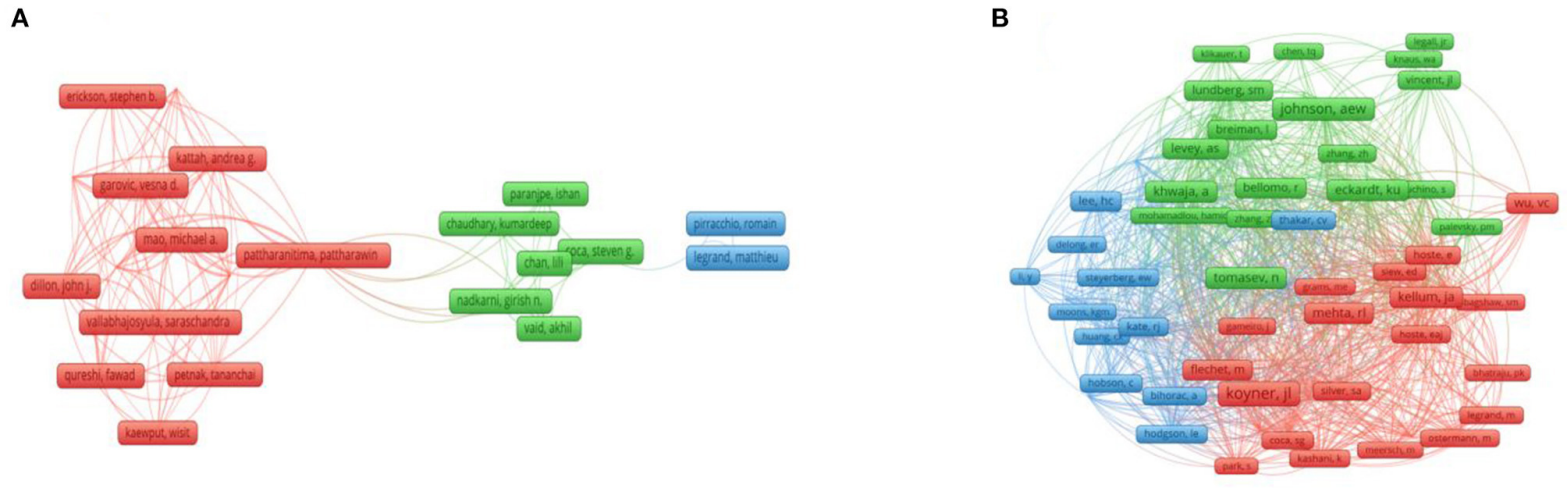
**(A)** Cooperation network of authors. **(B)** Co-citation network of authors.

The majority (83.7%) of all articles were produced by teams involving ≥3 authors. Table 2 shows that Bihorac, A and Ozrazgat-Baslanti, T from the University of Florida, USA, are the most productive authors. They both published 10 articles with a total of 280 citations and an H-index of 6. In addition, Baker, CR has the highest total number of citations (369), while Pattharanitima cooperated closely with Mao, MA, Dillon, JJ, and Cheungpasitporn, W (Figure 5A). In the co-citation network (Figure 4B), the top five co-citations were Koyner, JL (92), Johnson, AEW (71), Tomašev, N (64), Khwaja, A (63), and Kellum, JA (61) (Figure 5B).

**Table 2:** Top 10 authors by publications.

**Rank**	**Author**	**Publications**	**First**	**Correspond**	**Total citations**	**H index**
1st	Bihorac, A	10	1	8	280	6
2nd	Ozrazgat-Baslanti, T	10	1		280	6
3rd	Pattharanitima, P	8	3	2	35	4
4th	Liu, M	8		1	16	3
5th	Luo, Y	7		3	113	5
6th	Wu, VC	7	1	5	49	3
7th	Hu, Y	7		4	10	2
8th	Zhang, XZ	7			10	2
9th	Rashidi, P	6			219	4
10th	Nadkarni, GN	6		4	106	4

### 3.4. Journal analysis

Among all 185 journals, a total of 62 (33.3%) were classified as Q1 or Q2, including 10 journals with more than 5 articles. The five most prolific journals were Scientific Reports (IF4.996), Frontiers in Medicine (IF5.058), Journal of Clinical Medicine (IF4.964), BMC Medical Informatics and Decision-Making (IF3.298), and International Journal of Medical Informatics (IF4.730; Table 3).

**Table 3:** Top 10 journals by publications.

**Rank**	**Journal**	**Publications**	**Total citations**	**IF**	**5-year IF**
1st	Scientific Reports	19	106	4.997	5.516
2nd	Frontiers in Medicine	13	27	5.058	5.493
3rd	Journal of Clinical Medicine	13	214	4.964	5.098
4th	BMC Medical Informatics and Decision Making	10	163	3.298	3.894
5th	International Journal of Medical Informatics	8	188	4.73	5.076
6th	Critical Care	7	293	19.344	14.082
7th	JAMA Network Open	7	101	13.36	13.312
8th	Journal of the American Medical Informatics Association	7	130	7.942	7.041
9th	Plos One	7	161	3.752	4.069
10th	Frontiers in Cardiovascular Medicine	6	5	5.848	6.221

### 3.5. Cluster analysis of co-occurrence keywords

**Figure 6:**
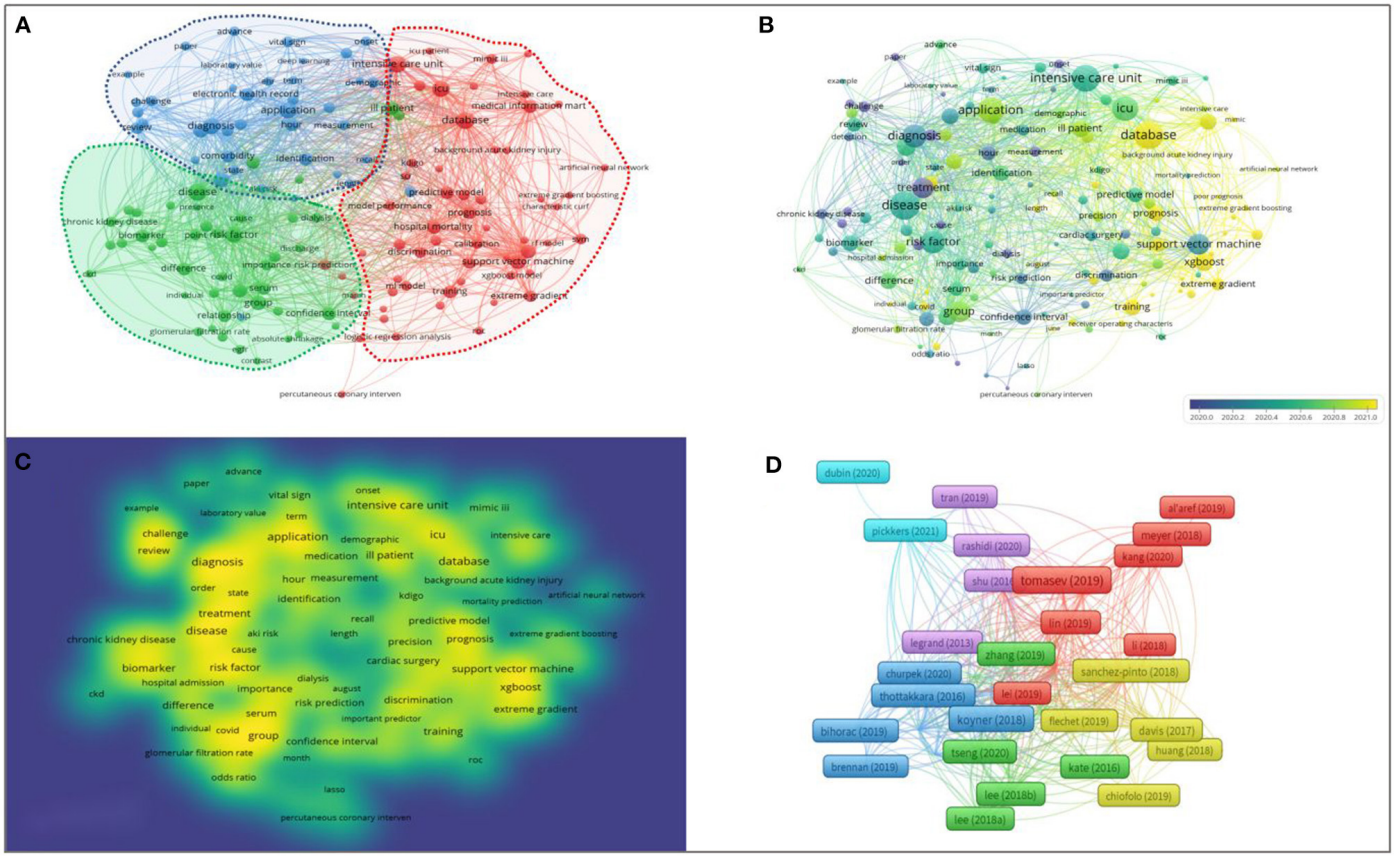
**(A)** Visualization map of co-occurring keywords. **(B)** Overlay map of keywords. The closer to blue the keyword node color, the earlier the time. **(C)** Density map of keywords. **(D)** Literature coupling analysis network map.

A map was then created by VOSviewer with 142 terms (8,259 in total), with at least 10 appearances per term (Figure 6A). Terms with comparable studies were merged under the same catalog with three main categories. The major red cluster #1 consisted of 58 terms, including “decision tree”, “random forest”, “XGBoost”, “Support Vector Machines”, “Extreme gradient”, and “MIMIC III”, which mainly focused on the algorithm study of machine learning-based AKI prediction models and highlighted the characteristics of modeling source data. The major green cluster #2 consists of 44 terms, including “severity”, “mortality”, “dialysis”, and “risk factors”, which mainly focused on the prediction results of the machine learning-based AKI prediction model, and the major blue cluster #3 consists of 40 terms, including “electronic health record”, “diagnosis”, “differentiation”, “treatment”, “demographic characteristics”, and “laboratory test value”, which mainly focuses on the characteristics of the included variables and the purpose of the machine learning-based AKI prediction model. The time overlay visualization indicated that “prognosis,” “sepsis,” “critically ill patients,” “MIMIC,” and “XGBoost” dominated in recent research (Figure 6B). In addition, the keyword density visualization indicates that “support vector machine”, “risk factors”, “diagnosis”, and “critically ill patients” still occupy the core part (Figure 6C).

### 3.6. Analysis of highly cited and co-cited literature

The top 10 highly cited studies on machine learning-based AKI research are shown in Table 4. These studies are mainly focused on machine learning-based AKI prediction models. The most notable is a report published in Nature by Tomašev et al.'s team in collaboration with Deepmind, a Google company, in which a model developed using deep learning recurrent neural networks based on over 700,000 case data provided by the U.S. Department of Veterans Affairs achieved an AUC value greater than 0.9 for predicting AKI events 48 h in advance, which is considered one of the most successful studies in the field of machine learning modeling to date.

**Table 4:** Top 10 highly cited literature.

**Rank**	**Title**	**First author**	**Source**	**IF**	**Year**	**Citations**
1st	A clinically applicable approach to continuous prediction of future acute kidney injury	Tomašev, N	Nature	69.504	2019	342
2nd	The development of a machine learning inpatient acute kidney injury prediction model	Koyner, JL	Critical Care Medicine	9.296	2018	136
3rd	Machine learning for real-time prediction of complications in critical care: a retrospective study	Meyer, A	Lancet Respiratory Medicine	102.64	2018	122
4th	Comparison of variable selection methods for clinical predictive modeling	Sanchez-Pinto, LN	International Journal of Me-dical Informatics	4.73	2018	100
5th	MySurgeryRisk: development and validation of a machine-learning risk algorithm for major complications and death after surgery	Bihorac, A	Annals of Surgery	13.787	2019	98
6th	Machine learning for the prediction of volume responsiveness in patients with oliguric acute kidney injury in critical care	Zhang, ZZ	Critical Care	19.344	2019	92
7th	Application of machine learning techniques to high-dimensional clinical data to forecast postoperative complications	Thottakkara, P	PLoS One	3.752	2016	90
8th	Calibration drift in regression and machine learning models for acute kidney injury	Davis, SE	Journal of the American Medical Informatics Association	7.942	2017	89
9th	Prediction and detection models for acute kidney injury in hospitalized older adults	Kate, RJ	BMC Medical Informatics and Decision Making	3.752	2016	87
10th	Prediction of acute kidney injury after liver transplantation: machine learning approaches vs. logistic regression model	Lee, HC	Journal of Clinical Medicine	4.964	2018	79

Among the top 10 highly co-cited references, four were practical guidelines and epidemiological analyses of AKI, indicating that scholars attach great importance to the basic pathogenesis of AKI, and four were studies of AKI predictive models, indicating that scholars are interested in the construction of AKI prediction models. The remaining three articles examined the introduction of the GRADE system, the TRIPOD initiative for prediction model studies, and the introduction of the MIMIC database, which shows that scholars attach great importance to modeling research methods and the details of article writing and publication (Table 5).

**Table 5:** Top 10 co-citation references.

**Rank**	**Title**	**First author**	**Source**	**IF**	**Year**	**Citations**
1st	A clinically applicable approach to continuous prediction of future acute kidney injury	Tomašev N	Nature	69.504	2019	64
2nd	KDIGO Clinical Practice Guidelines for Acute Kidney Injury	Khwaja A	Nephron Clinical Practice	2.138	2012	63
3rd	Foreword	Eckardt KU	Kidney International Supplements	6.083	2012	59
4th	MIMIC-III a freely accessible critical care database	Johnson AEW	Scientific Data	8.501	2016	59
5th	The Development of a Machine Learning Inpatient Acute Kidney Injury Prediction Model	Koyner JL	Critical Care Medicine	9.296	2018	50
6th	Transparent Reporting of a Multivariable Prediction Model for Individual Prognosis or Diagnosis (TRIPOD): The TRIPOD Statement	Collins GS	European Urology	24.344	2015	37
7th	Epidemiology of acute kidney injury in critically ill patients: The multinational AKI-EPI study	Hoste E	Intensive Care Medicine	41.787	2015	36
8th	Prediction detection models for acute kidney injury in hospitalized older adults	Kate RJ	BMC Medical Informatics Decision Making	3.298	2016	35
9th	AKIpredictor an online prognostic calculator for acute kidney injury in adult critically ill patients: Development validation comparison to serum neutrophil gelatinase-associated lipocalin	Flechet M	Intensive Care Medicine	41.787	2017	34
10th	Acute Kidney Injury Network: report of an initiative to improve outcomes in acute kidney injury	Mehta RL	Critical Care	19.344	2007	30

In addition, in the literature coupling analysis network contribution map (Figure 6D), it was found that Tomašev et al.'s “A clinically applicable approach to continuous prediction of future acute kidney injury” in 2019, Meyer et al.'s “Machine learning for real-time prediction of complications in critical care” in 2018, and Koyner et al.'s “The Development of a Machine Learning Inpatient Acute Kidney Injury Prediction Model” in 2018 had higher centrality values. In contrast to the high citation results, this result suggests that these three papers have relatively comprehensive and authoritative reference citations, suggesting that these three papers have better generalization and summary for machine learning-based AKI studies.

### 3.7. Analysis of funding agencies

The top five funding sources supporting the highest number of publications are the United States Department of Health Human Services (HHS), National Institutes of Health (HIH), National Natural Science Foundation of China (NSFC), National Institute of Diabetes Digestive Kidney Diseases (NIDDK), and National Center For Advancing Translational Sciences (NCATS). Nine of the top ten funding agencies are from national or other public organizations, including a total of seven from the United States, representing the emphasis of national research programs on machine learning-based AKI research (Table 6).

**Table 6:** Top 10 funding sources by publications.

**Rank**	**Funding source**	**Publications**	**Country/region**
1st	United States Department of Health Human Services	63	USA
2nd	National Institutes of Health	61	USA
3rd	National Natural Science Foundation of China	42	China
4th	National Institute of Diabetes Digestive Kidney Diseases	21	USA
5th	National Center for Advancing Translational Sciences	19	USA
6th	National Science Foundation	13	USA
7th	National Institute of General Medical Sciences	13	USA
8th	European Commission	11	Europe
9th	Ministry of Science and Technology Taiwan	10	Taiwan
10th	National Library of Medicine	8	USA

## 4. Discussion

In recent years, studies on AKI have been gradually enriched, but the main direction of clinical research is still limited to epidemiology, risk factors, and prognosis. Scholars are urgently seeking breakthroughs in new research directions to achieve innovation in AKI diagnosis and treatment models, and the emergence of machine learning methods has added a new highlight to the “research anxiety” of scholars. In the past decade, the publication on the study of machine learning-based AKI research has gradually increased, to further predict the hotspots precisely and make suggestions for the future perspective, we finally utilized bibliometric technology to analyze the latest literature in this field between 2013 and 2022 globally, providing a basic reference for scientists to discover the hotspots and frontiers.

We retrieved more than 300 related studies based on the Web of Science database, and according to our selected narrower research target direction, this result is encouraging. In addition, according to the year analysis, we found that the volume of literature in the last 2 years occupies more than 2/3 of all published literature, which is an indication of the new hotness of this research field. The results are encouraging, and along with the investment of more resources, this research field will also gain more momentum.

As the main driver of research, the contributions of the United States and China in this field are evident. Many institutions from the United States have carried out a large number of studies and have outstanding advantages in terms of published literature, citations and H index, but the research of Chinese scholars in this area is equally exciting, as it is well-known that China has a high incidence and unrecognized rate of AKI. In a study in 2013 (13), it was estimated that the number of AKI patients in China throughout the year was approximately 1.4–2.9 million, of which the unrecognized rate was approximately 97–99%. This background provides the premise and necessity for the study of AKI, especially for the study of prediction models based on machine learning. However, Chinese research is equally innovative; for example, in a 2009 study by Zhang et al. (14), the potential of machine learning methods to distinguish volume reactive and volume non-reactive AKI was successfully demonstrated. European countries such as the Netherlands, the United Kingdom and France also conduct similar studies, but the cooperation between countries or institutions, including the United States, is not close. On the one hand, this has limited the development of studies on machine learning-based AKI research. On the other hand, the reliability of prediction models for transcentric migration cannot be verified.

In terms of author contributions, both prolific authors published 10 papers, while Bihorac, Azra and Ozrazgat, Baslanti, Tezcan, both from the University of Florida, USA, had the highest H-index and high total citations, and their research focused on the prediction of surgery-related AKI (15–17). Liu et al. preferred to predict critical AKI patients (18, 19) and focused on exploring the variable selection and time window settings in machine learning prediction models (20). Although the overall evaluation index of published literature is not dominant, it is also critical for the guidance of future scholars. In addition, Tomašev has only three related publications, but its “A clinically applicable approach to continuous prediction of future acute kidney injury” published in Nature has the highest total citations. The study, which was a collaboration with DeepMind, a Google company, uses a deep learning approach to build a real-time prediction model for AKI and is considered a representative work in this field (21). In addition, in the co-citation network, it was found that the study by Koyner et al. (22). was referenced by more peer literature, and the predictive efficacy of machine learning models for predicting creatinine change in patients with AKI was confirmed earlier in one of their 2018 studies, and in the coupling analysis of references, it was found that this literature, like the abovementioned literature by Tomašev et al., had a higher centrality value, this means that the references of these literatures are cited by other similar research institutes at the same time, indicating that their references are more authoritative and comprehensive, and scholars can better understand the research basis of this field by searching and reading these references.

The highly cited literature on machine learning-based AKI mainly focuses on modeling studies, which are consistent in terms of the number of cases included in the study cohort and the selection of machine learning methods. Among the top ten ranked studies, Zhang et al.'s study in 2019 included the smallest sample size, which still exceeded 6,000 cases (14), while Tomašev et al.'s study even exceeded 700,000 cases (21), which again proves the preference and applicability of machine learning for large sample size data and encourages future researchers to place more emphasis on sample size. However, it must be noted that the endpoint events of the current study are more focused on the AKI outcome of all-cause inpatients, while in studies of AKI prediction models related to specific comorbidities (23), specific nephrotoxic drugs (24), and specific procedures (25), the selection of sample size is influenced by morbidity, and given the scarcity of similar peer studies, the published literature of such studies is often underestimated. These highly cited studies also differed in terms of predictive endpoint events, model type, and predictive timeliness, with several studies using AKI onset as the predictive outcome and 48 h earlier as the timeliness assessment point (21, 22), but some studies have developed a real-time prediction model considering the temporal changes in AKI events (26, 27). In addition, the analysis revealed that studies on variable selection and model performance calibration were equally valued (28), especially this study still occupying the eighth position in the analysis of co-cited references, suggesting that the issues raised and addressed in this study may be generalized across all modeling studies and that future researchers read these two papers as a way to avoid repeating the same mistakes in their methodological design.

The top 10 co-cited references included practical guidelines and epidemiological analysis of AKI (29–31), AKI modeling (21, 22, 32, 33), TRIPOD initiative for predictive modeling studies (34), introduction of MIMIC database, and GRADE system (35, 36), which shows that researchers attach great importance to the pathogenesis of AKI, study process design, and literature writing, especially Collins et al. (34) proposed the TRIPOD initiative of prediction model research report in view of the poor quality of the current prediction model report. This initiative supports any model report to be accompanied by a detailed explanation and elaboration to describe all aspects of the research and emphasize inappropriate practices that should be avoided. Additionally, the initiative lists 22 inspection items that should be included in the model report to help the author conduct literature self-inspection, peer reviewers review and interested people better critically read the published literature.

The cluster analysis of high-frequency keywords and references can identify the hot spots and frontiers of machine learning-related AKI research, among which “prognosis”, “sepsis”, “critically patients”, “MIMIC”, and “XGBoost” are the hot spots of research based on the time overlay map. The current research on machine learning in sepsis-related AKI mainly includes two aspects: one is the study of constructing predictive models (37), and the other is the auxiliary addition to the analysis and processing of ultrasound images of AKI (8, 38). In several studies, the diagnosis and treatment effect of sepsis-related AKI has been greatly improved by introducing XGBoost, deep learning and neural network algorithms (8, 37, 38), but its effectiveness in clinical practice remains to be confirmed. HA-AKI in critically ill patients has always been one of the research hotspots in this field (39–42). The performance of the models reported thus far can achieve more than moderate discrimination of AKI events, but they still have some shortcomings, including the lack of effective external validation of the model, the lack of model interpretation, and the differences in the inclusion of model variables. The selection of datasets for model development should also be more extensive, while not rejecting open source datasets such as MIMIC-III and AmsterdamUMCdb, more reference should be made to local datasets to achieve generalizability testing of the model in different populations, which is also necessary for cross-center migration of the model.

Combined with the above analysis, there are also several suggestions for the future development of machine learning-related AKI research: (1) insisting on an adequate sample size of study cohorts may be necessary to realize the potential of machine learning; (2) based on the predictive effect of novel biomarkers for subclinical AKI, introducing novel biomarkers as effective variables may further improve model performance, which is extremely rare in the current research; (3) developing predictive models for nephrotoxic drug-related AKI based on specific populations of contrast and chemotherapeutic drug use is also of great interest; in particular, the reports of immune checkpoint inhibitor AKI have gradually increased in recent years, while such studies are still not reported in the literature; and (4) it is necessary to conduct a prospective external validation study of the model, and more randomized controlled trials (RCTs) are called for in this field to further support the research conclusions.

Our study also has some limitations. First, because it is difficult for bibliometric analysis software to analyze data from multiple databases, only the WoSCC database was included in our study, but data from Scopus, PubMed, and CNKI were also needed in the comprehensive analysis. Second, we only included English literature and did not include literature reports in other languages, especially Chinese reports, which have been on the rise in recent years, so we may have missed some research hotspots.

## 5. Conclusions

Based on the bibliometric analysis, we explored the current status of publications on machine learning-based AKI research in the past decade. At present, the number of publications in this field is increasing year by year, among which the United States and China make the greatest contribution; moreover, national institutions give considerable support to this type of research. Relevant scholars have relatively unique research directions and special preference for some journals. Keyword clustering analysis suggests the current stage of research hotspots, and the analysis of highly cited and co-cited literature provides the best reference for new entrants to peer research, but it is worth noting that there is still a lack of effective collaboration between different countries, institutions, and independent researchers, which is crucial for the continued development of research in this field.

## Data availability statement

The original contributions presented in the study are included in the article/supplementary material, further inquiries can be directed to the corresponding author.

## Author contributions

All authors listed have made a substantial, direct, and intellectual contribution to the work and approved it for publication.
