# Corrigendum: Bibliometric and visual analysis of machine learning-based research in acute kidney injury worldwide

**DOI:** 10.3389/fpubh.2024.1430491

**Published:** 2024-06-11

**Authors:** Xiang Yu, RiLiGe Wu, YuWei Ji, Zhe Feng

**Affiliations:** ^1^State Key Laboratory of Kidney Diseases, Department of Nephrology, Chinese People's Liberation Army General Hospital, Chinese People's Liberation Army Institute of Nephrology, National Clinical Research Center of Kidney Diseases, Beijing, China; ^2^Medical Big Data Research Center, Chinese People's Liberation Army General Hospital, Beijing, China

**Keywords:** machine learning, acute kidney injury, bibliometric analysis, model, critical care, hotspot

In the published article, there was an error.

“Regarding authors, Bihorac, A and Ozrazgat-Baslanti, T from the Kansas City Medical Center have published 10 articles.”

“Table 2 shows that Bihorac, A and Ozrazgat-Baslanti, T from the Kansas City Medical Center, USA, are the most productive authors.”

“In terms of author contributions, both prolific authors published 10 papers, while Bihorac, Azra and Ozrazgat, Baslanti, Tezcan, both from the University of Gainesville School of Medicine, USA, had the highest H-index and high total citations, and their research focused on the prediction of surgery-related AKI (15–17).”

The corrected sentence appears below:

“Regarding authors, Bihorac, A and Ozrazgat-Baslanti, T from the University of Florida have published 10 articles.”

“Table 2 shows that Bihorac, A and Ozrazgat-Baslanti, T from the University of Florida, USA, are the most productive authors.”

“In terms of author contributions, both prolific authors published 10 papers, while Bihorac, Azra and Ozrazgat, Baslanti, Tezcan, both from the University of Florida, USA, had the highest H-index and high total citations, and their research focused on the prediction of surgery-related AKI (15–17).”

The authors apologize for this error and state that this does not change the scientific conclusions of the article in any way. The original article has been updated.

